# Botulinum Toxin: Surely, We Can Do Better? Optimizing Results Beyond On-Label Techniques and Teaching

**DOI:** 10.1093/asjof/ojaf032

**Published:** 2025-04-30

**Authors:** Greg J Goodman, Adrian Lim, Sarah Hart, Peter Callan, Sarah G Boxley, Cara B McDonald, Frank Lin, Michael Clague, Niamh Corduff, Stefania Roberts, Alice Rudd, Katy Wallace, Narendra Kumar, Antoinette Ciconte, Firas Al-Niaimi, Sean Arendse, Philip Bekhor, Lee-Mei Yap, Maeve A Ahern, Andrew Clark, Anita Patel, Terence Poon, Howard M Studniberg, Catherine E Porter, Aakriti Gupta, Nina Wines, Linda Williams

## Abstract

Facial expressions and their emotional attributes are essential as an adjunct to verbal communication and for nonverbal communication. Botulinum toxin (BoNTA) used to limit wrinkles induced by aging changes on expressive faces. However, suboptimal injection techniques can lead to undesirable outcomes, such as brow ptosis, unnatural eyebrow elevation, flattening of the cheeks, and unnatural smiles. It is the intention here to investigate whether “on-label” standard injection techniques may be a contributing factor to poor results. A consensus group convened in 2023 looked at the relevant anatomy, expressions induced, complications of on-label BoNTA treatment, solutions, and alternative treatments. Interrogation of databases was supplemented by expert opinion of the consensus group. The authors of this review found that anatomical variations, generic on-label injection patterns, and inadvertent muscle recruitment contribute to suboptimal outcomes. Undesirable outcomes included brow ptosis, unnatural eyebrow shape, smile pattern alteration, and cheek flattening. These were linked to on-label standard protocols that fail to address individual anatomy and functional variations. Adjustments in dose, depth, and injection placement, along with simultaneous treatment of linked or antagonistic muscles, were identified as critical solutions. To optimize outcomes, it is critical to tailor injection strategies respecting individual anatomical and functional differences. Many unnatural appearances seen after BoNTA seem to have their origin in on-label techniques suggested by manufacturers. Although on-label protocols have been useful as a guide, the authors feel it is time to move past these to account for patient-specific needs and anatomical nuances. In this review, the authors provide practical recommendations for improved injection techniques.

**Level of Evidence:** 5 (Therapeutic)

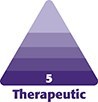

Since ancient times, nonverbal communication has preceded verbal communication.^1^ Facial expressions act as important cues for social communication, conveying feelings of anger, sadness, surprise, happiness, contempt, skepticism, or disappointment.^2,3^ Although botulinum toxin treatments improve negative facial expressions such as frowning or a downturned mouth, unintended consequences can also arise. These include a false smile that does not reach the eyes; overarched brows creating an evil Mephistopheles look; low, flat brows denoting sadness; and excess upper eyelid skin giving a tired appearance. Aesthetic practitioners can unintentionally change facial muscle activity, altering facial communication and impacting our patients professionally, socially, and economically.^4^

Botulinum neurotoxin A (BoNTA) is a powerful tool for aesthetic practitioners, enabling the intentional reshaping of facial expressions, such as softening angry or sad appearances. However, it is equally capable of producing undesirable side effects if not used with precision and a deep understanding of facial anatomy.^5^

The combination of suboptimal anatomy knowledge, regimental on-label training techniques promoted by the manufacturers, and inadvertent muscle recruitment were hypothesized to lead to these less-than-desirable outcomes. It was further considered that teaching on new or existing products may be centered on “what has gone before” copying comparator injection patterns in previous registration studies, probably oblivious to the ingrained issues with these patterns.

## METHODS

A consensus group convened in March 2023 at the annual meeting of the Australasian Society of Cosmetic Dermatologists to investigate suboptimal aesthetic outcomes of BoNTA injections. The group reviewed the complications, training techniques, and treatment recommendations. The group decided to consider the reasons for the commonly seen poor aesthetic outcomes and make appropriate recommendations. This consensus concentrates on the upper face, examining BoNTA use in the forehead, glabella, periorbital lines, and nose.

The consensus group was composed of 28 practitioners with varying backgrounds, including plastic and ophthalmic surgeons, dermatologists, aesthetic general practitioners, nurse practitioners, and nurses.

The process for consensus was to divide the consensus group into working parties with responsibilities to write sections of the manuscript. Initial literature searches were conducted in April 2023. These sections were the forehead (frontalis), the frown (glabella complex), bunny lines, and “crow's feet” (the role of orbicularis oculi [OOc]). The individual group’s initial writings were scrutinized by the entire consensus group through 2 Delphi style reviews and then assembled into the final manuscript. Disputes were handled with further examination of available literature and more review by the full consensus group. There were no disputes that required voting by the consensus group, with unanimous agreement on all aspects of the paper being achieved.

The experts investigated each region as outlined below:

1. The targeted expression and assessment
2. Relevant muscular anatomy of the region
3. Variations in movement patterns
4. On-label or standard techniques
5. Problems that may arise
6. Possible solutions
7. Alternative treatment options

An extensive literature search was conducted using databases, such as Google Scholar, PubMed, Embase, and Scopus, to find supporting evidence. However, in addition to considering scientific evidence, the group's views were considered in the context of changing trends and fashion.

## RESULTS

### The Forehead (Frontalis)

#### The Targeted Expression and Assessment

BoNTA injections into the frontalis muscle have been used to reduce hyperdynamic forehead rhytids for nearly 30 years.^6^ Contraction of the frontalis muscle raises the brows and upper eyelids and can pull down the hairline, producing horizontal forehead lines (Supplemental Tables 1-3).^7^ Therefore, significantly weakening the frontalis muscle to reduce the horizontal forehead lines may cause undesirable aesthetic and functional outcomes, such as brow ptosis.^8^ Efforts to minimize the risks of brow ptosis by partially treating the frontalis muscle can lead to uneven muscle immobilization and excessive recruitment of unrelaxed muscle fibers with the potential to create a range of undesirable aesthetic outcomes.^9,10^

The consensus group agreed that many anatomical variations of the frontalis muscle, coupled with its complex interplay with the antagonistic brow depressors, make the treatment of this region challenging.

#### The Relevant Muscular Anatomy of the Frontalis Muscle

The frontalis muscle originates from the galea aponeurotica and inserts into the surrounding soft tissue of the brow, the procerus, corrugator supercilii, and OOc muscle. Evidence suggests that in over 87% of cases,^11^ the frontalis muscle has 2 bellies. Frontalis has no bony origins but rather arises from the aponeurosis and inserts into subcutaneous fat and the dermis of forehead skin.^12^ Moreover, sometimes its lateral border inserts into the superior temporal septum (Supplemental Tables 1-3).^13,14^

Muscles of the glabella complex and the superior-lateral OOc oppose the elevating action of the frontalis muscle, depressing the position of the brow.^12^ BoNTA injections into these depressor muscles help regulate the position of the brow relative to the eyes by reducing the opposing pull of the frontalis muscle.^15^

In 1 article, distinct segments of the frontalis muscles have been suggested to exist: a lower segment responsible for raising the eyebrows and an upper section that lowers the hairline. These segments meet at a horizontal line on the forehead described as the “line of convergence” or “C-line,” located at ∼60% of the total height of the forehead, and remain consistent across genders and ethnicities.^16^

This remains a conjectural concept, and some feel this has as much to do with tone and tissue thickness and turgor when a mobile structure is being pulled from different directions.

It is vital to understand the variation in the anatomy of the frontalis muscle, and its antagonistic muscles. Moreover, it is essential to understand how the interplay between these muscles affects the function and appearance of the patient before injecting BoNTA into the frontalis muscle to reduce these horizontal lines.^9^

Aging of the upper face and its manifestation, such as an increase in the aperture of the bony orbit, loss of fat support under the skin, and increased tonicity of the brow depressor muscles, combine to contribute to brow ptosis with or without upper lid dermatochalasis.^17^ These changes may, in turn, obstruct the upper outer quadrant of the visual field,^18^ resulting in compensatory activation of the frontalis in many patients who seek treatment to improve the cosmetic appearance of the forehead. Assessment requires observation of the brow position at rest and on animation. By paying attention to the extent of movement, severity, and location of the rhytids in static and dynamic states, it is possible to estimate the shape and boundaries of the frontalis muscle.

#### Variations in Movement Patterns

Clinically, there is true variation in frontalis-induced forehead lines. Four common patterns^18^ have been described in the literature (Table 1).

**Table 1.** Patterns of Forehead Lines

Patterns of forehead lines
Full, straight lines across the whole forehead (45%)
2. Wing-shaped lines with a central depression and lateral elevation (30%)
3. Short central horizontal lines over the middle, but few or no lines laterally (10%)
4. Lateral straight lines as 2 columns formed on the lateral aspect of the forehead with no central lines (15%)

#### On-Label Techniques

**Figure 1.**
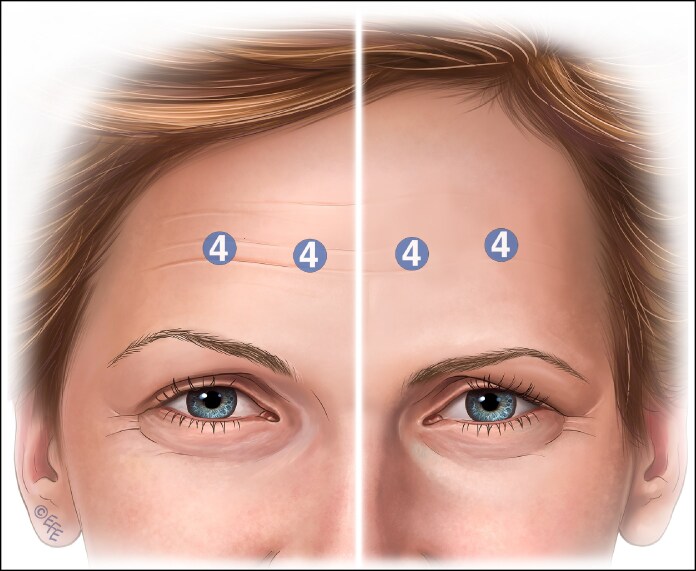
Four injection points showing injection points and on-label suggested onabotulinum toxin units. Brow position before injection on the right side of the figure and the possible brow ptosis after botulinum injection shown on the left side. This is particularly likely in an older individual with less structural support.

The recommended standard treatment technique is 4 injection points equally spaced across the frontalis in a horizontal pattern midway between the brow and the hairline, more recently scientifically referred to as the C-line. However, a single injection pattern for every patient fails to address individual anatomical variations and leads to poor outcomes.^19-21^ In Figure 1, brow ptosis may result from the depressor effect of an injection that fails to account for the specific anatomy of the individual or the nuanced needs of an older patient.

#### The Problems and How They Arise

On-label BoNTA injection patterns sparing the inferior frontalis may result in overcompensatory activation of these fibers, causing accentuation of previously unnoticed wrinkles, often coupled with aesthetically unpleasing lateral brow elevation.^22^

Facial asymmetries are relatively common and well documented in the literature. Studies have reported over 88% of the population have asymmetric brow positions, which need to be accounted for during any kind of surgical or nonsurgical interventions.^23,24^ Five percent of patients, who were treated with botulinum toxin injections as per suggested protocols in the package inserts, require further touch-ups to correct the eyebrow asymmetry.^9^ Therefore, it is mandatory to observe the eyebrow positions and contraction pattern of the frontalis muscle during the assessment; accordingly, individualized BoNTA doses and injection points should be determined. Such practice will minimize the occurrence of posttreatment brow asymmetry and poor aesthetic outcomes (Table 2).

**Table 2.** Origins and Insertions of the Frontalis Muscle

Origin	Insertion	Nuances of origin	Nuances of insertion
Galea aponeurotica	Soft tissues surrounding the brows, including the procerus, corrugator supercilii, and orbicularis oculi^9,11^	Two heads of frontalis in 87% of cases^11^	No bony insertions Connections to subcutaneous fat and into the dermis of forehead skin^12^ The lateral border of frontalis insertion is usually but not always at the superior temporal septum^13,14^

Skin quality and existing static forehead rhytids are closely linked to treatment satisfaction and perceived longevity of the toxin in these patients. Thin, crepey, sun-damaged skin combined with a loss of fat in the forehead reduces the success of BoNTA treatment in the frontalis. Injectors need to understand that with frontalis, the skin quality is vital to the success of the BoNTA result (both initially and over the 4 months).

#### Possible Solutions

Assessment and individualization of BoNTA dose, depth, and injection points are critical for optimal aesthetic outcomes. Practitioners should be prepared to review the patient and adjust the dose. Additionally, there is a need to be mindful of the reciprocal relationships between dose and longevity and the balance between dose and effect.

When patients are asked to elevate their eyebrows, they may or may not depress their hairline with this effort. For those who depress their hairline, they will likely need an injection of the botulinum toxin along their hairline to prevent these upper lines from being left after treatment. This is less likely to be an issue for those who do not lower their hairline when elevating their eyebrows.

It is also important to treat the brow depressors simultaneously with frontalis treatment. This may help to avoid brow ptosis, as unopposed action by the brow depressors once frontalis elevation is inactivated may exacerbate the tendency to brow ptosis.

Depth of injection may impact the aesthetic outcome. Recent clinical studies have demonstrated that when compared with a deeper injection approach (=“intramuscular”) for treating horizontal forehead lines, a more superficial injection technique (=“intradermal”) can produce safer outcomes and prevent adverse effects (eyebrow ptosis).^25^ This suggests that the efficacy of neuromodulator therapies varies depending on the layer at which they are administered.^26^

**Figure 2.**
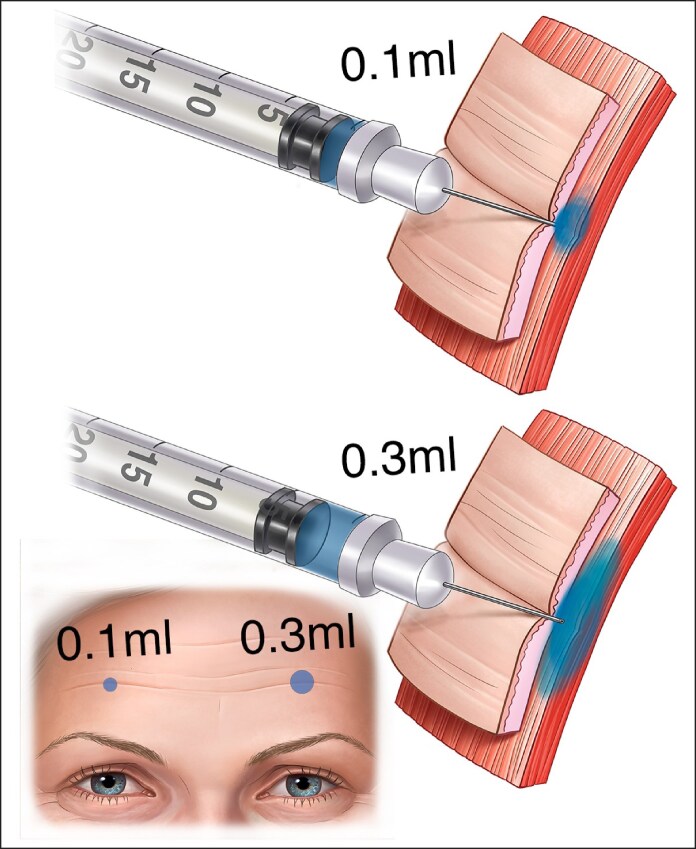
The same dose may be given by a more concentrated (here 0.1 mL) with less diffusion or more diluted resulting in a greater diffusion ring.

Moreover, within the range of now available toxins, it is vital to respect the nuances between toxins and the injection depth required for each toxin to be effective. Altering the diffusion of the neurotoxin in the frontalis is sometimes desirable. Hyperdilution of abobotulinum toxin with 0.3 vs 0.1 mL of saline induces higher diffusion in the forehead.^27^ An injector may consider a lower dilution volume to reduce diffusion and manage the accuracy of dose placement (Figure 2).^27^

#### Alternative Treatment Options

Energy-based devices, superficial threads, and laser-assisted drug delivery of growth factors may assist in thickening the dermis of the forehead. Another approach is to inject low G prime soft-tissue fillers into the superficial dermis, smoothing the lines. However, injectors must be aware of the high risk of serious intravascular accidents in this area.

### The Frown (Glabella) Complex

#### The Targeted Expression and Assessment

The glabellar complex plays a crucial role during verbal as well as nonverbal communication through a variety of expressions. The intention is to convey a variety of emotions depending on the context, including displeasure or disapproval, anger or annoyance, and concentration (Table 3).

**Table 3.** Glabella Frown Complex: Targeted Expression, Effect, and Muscle

Targeted expression	Effect	Targeted muscle
Frown	Inducing downward pull on medial eyebrows and mid-forehead	Procerus and depressor supercillii
Frown	Inducing medial pull on eyebrows	Corrugator supercilii and medial orbicularis oculi

#### Relevant Muscular Anatomy of the Glabella and Brow Position

**Figure 3.**
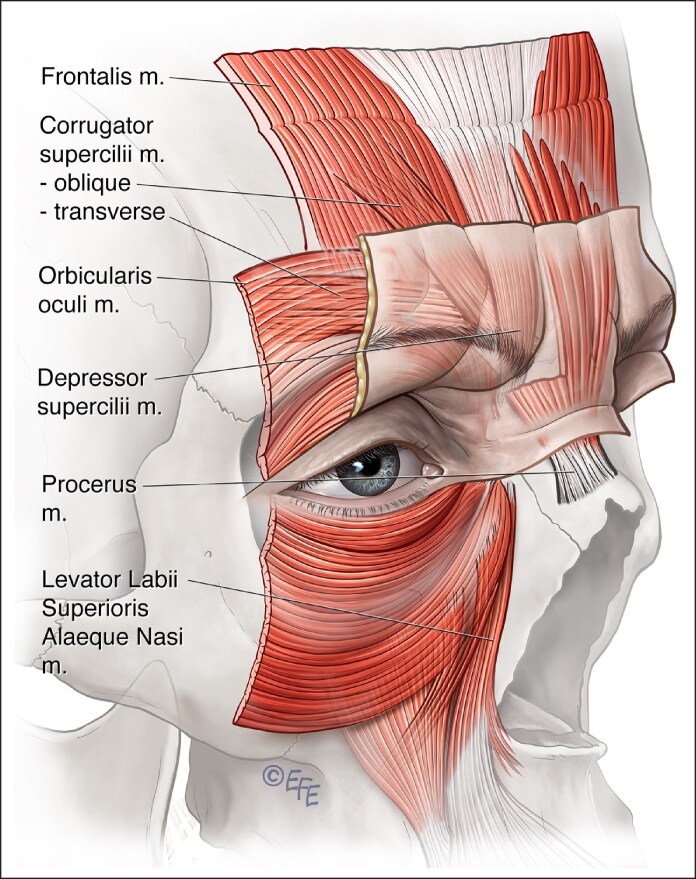
The muscles responsible for the appearance of frowning. The corrugator supercilii muscle originates at the medial end of the superciliary arch and has 2 heads, the oblique and the transverse. The transverse head is largely responsible for the frown and inserts laterally into the skin of the brow. The procerus muscle is a pyramidal-shaped muscle arising from the fascia of the superior nasal region near the junction of the nasal bones and the superolateral nasal cartilage. The procerus muscle fibers run superiorly and merge with the frontalis muscle. Muscle fibers insert into the skin between the eyebrows.

Glabellar frowning occurs from the combined coordinated action of the frontalis, corrugator supercilii, OOc, and procerus muscles (Supplemental Tables 1-3 and Figure 3).^28-31^ Moreover, several minor muscles, such as inferior-medial OOc, levator labii superioris alaeque nasi (LLSAN), and depressor supercilii, also play a role in determining the frowning pattern. Occasionally these minor muscles may be recruited during frowning and cause bunny lines and other expression oddities if not treated.

#### Variation in Movement Patterns

**Figure 4.**
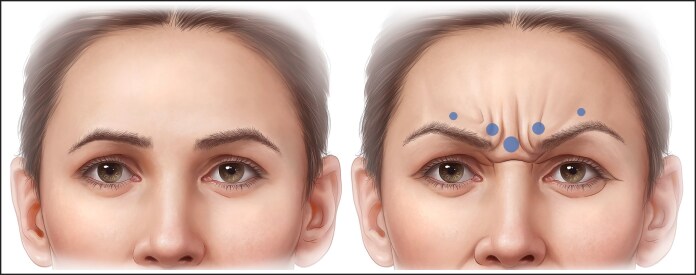
Example of a “U” pattern of contraction that may well be addressed using the standard 5-site injection scheme.

It is essential to assess each patient's frowning pattern to determine the required dose at each injection point. However, some patients find it challenging to frown on command and may display unfamiliar activity patterns. On-label teaching tends to predetermine dose and injection pattern; however, this is not used by most experienced injectors. de Almeida et al described 5 patterns of glabella lines V, U, omega, converging arrows, and inverted omega (Figure 4).^32^

#### On-Label Techniques

Standardized treatments of the glabella include a 3-, 5-, or 7-point injection technique.^33,34^ However, these guidelines are based on clinical trials for United States FDA registration purposes and do not reflect real-life practice. The standard recommendation is for a 5-point injection (Figure 4)—1 injection to the head and tail of each corrugator and 1 to the procerus—at a starting dose for incobotulinum toxin type A or onabotulinum toxin type A of 20 and 40 units for females and males, respectively.^35-38^ Abobotulinum toxin type A has a starting dose of 50 units, 10 units per injection point.^39^

#### The Problems and How They Arise

The frown complex usually involves 1 of 3 common suboptimal outcomes when injecting: the Mephistopheles effect, eyebrow splaying, and upper eyelid ptosis.

**Figure 5.**
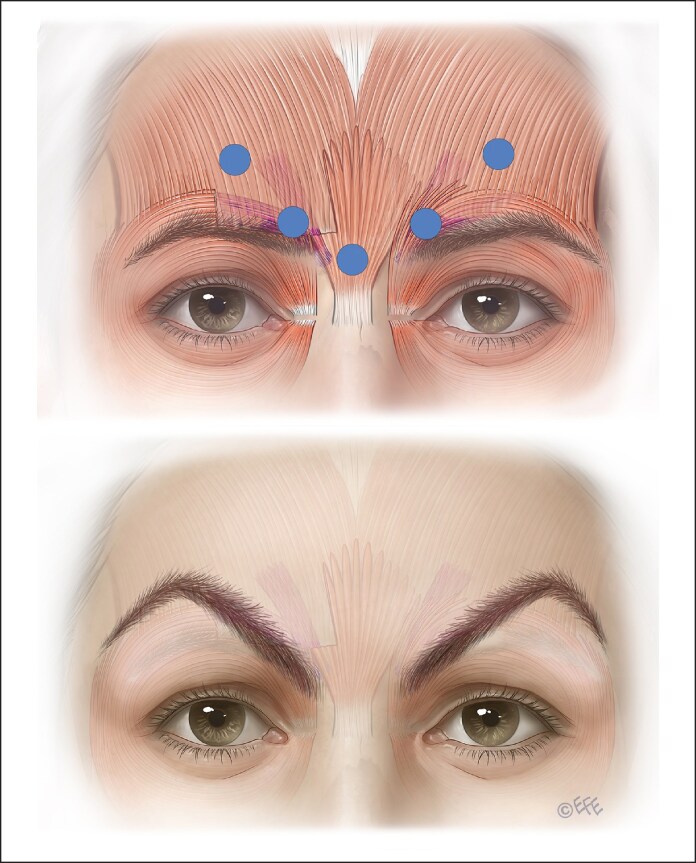
The Mephistopheles effect is caused by the high placement of medial and especially lateral injection points above the corrugator supercilii and targeting the frontalis resulting in medial brow depression and compensatory lateral brow elevation. This typically occurs when a practitioner follows the on-label advice of injecting at least 1 cm above the supraorbital rim at the lateral injection point but misinterprets this as 1 cm above the brow.

The Mephistopheles effect or “spock brows” describes eyebrows that upturn sharply and unnaturally. In this situation, the lateral end of the eyebrow is translocated above the medial end, producing a peculiar, unnatural-looking brow arch, or a quizzical look (Figure 5).

**Figure 6.**
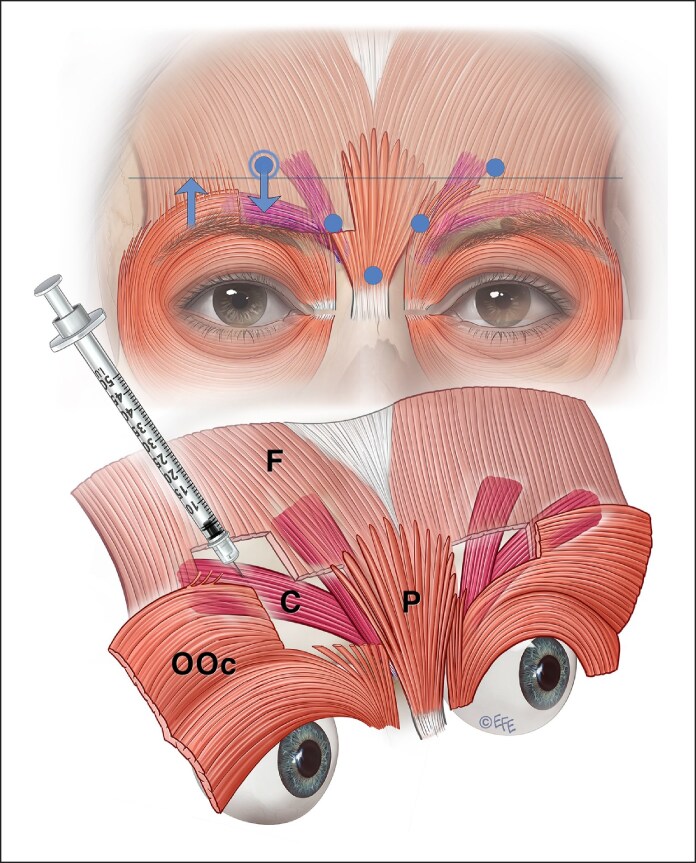
The upper part of this diagram shows a common issue with on-label botulinum injection. The diffusion circle will reach the corrugator but will most affect the medial frontalis. The second diagram shows the preferred lateral injection into the corrugator tail.

**Figure 7.**
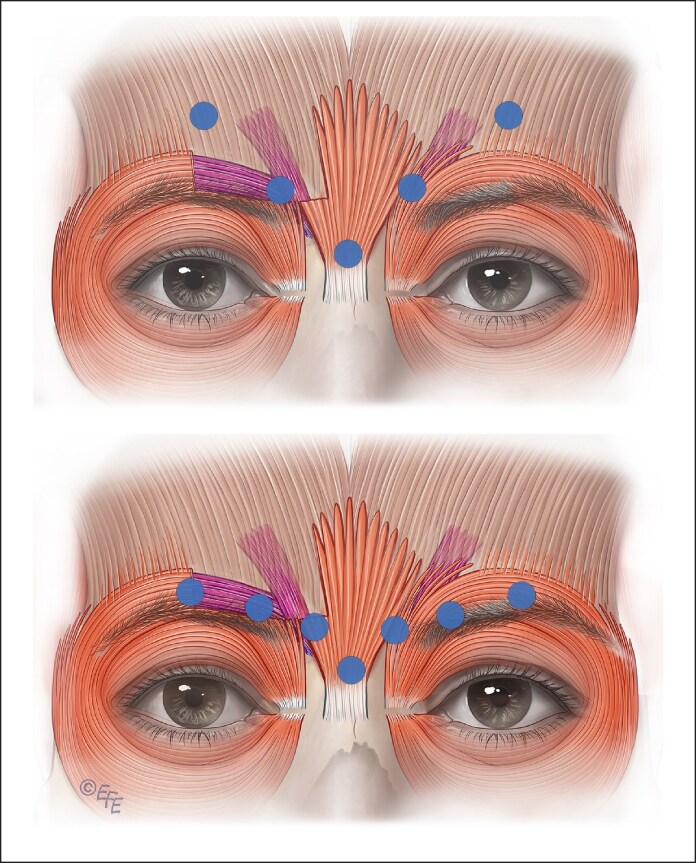
The upper diagram shows a common issue with on-label botulinum injection. The lower diagram illustrates a lower botulinum toxin delivery to better targeting the dose to the corrugator supercilii rather than the frontalis muscles. Contrary to traditional on-label injecting lower doses should be delivered to the lateral injection points to the tail of the corrugator.

**Figure 8.**
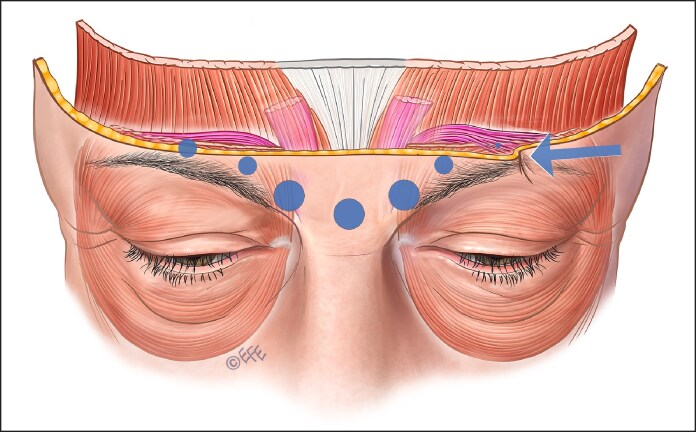
Incomplete lateral injection on the left of the final insertion points of the corrugator supercilii leading to residual action of corrugator supercilii laterally.

Current on-label recommendations emanated from manufacturers' registration trials (Figures 6-8), where study approval requires the lateral corrugator injection points to be placed well above the supraorbital rim to avoid eyelid ptosis from the spread of BoNTA into the levator aponeurosis from lateral injection points. It was also suggested that equal amounts should be injected in a 5-point technique (Figure 8).

However, this injection method primarily targets the frontalis muscle's lower fibers rather than the corrugator's tail. This results in depression of the medial brow because the only elevator of the brow (the frontalis) is being targeted, albeit unintentionally (Figures 6, 7). The relatively high dose laterally is arguably not required and adds to diffusion to the frontalis. Because most of the frontalis has not been injected, recruitment occurs and the tail of the brow is raised by lateral fibers of the frontalis, producing a peculiar, unnatural-looking arching of the brow.

The glabella widening is more likely to occur with complete inactivation of the corrugators and procerus with additional inactivation of the medial frontalis, leaving the lateral frontalis and OOc to pull the eyebrow laterally unopposed.

Upper eyelid ptosis is caused by the diffusion of the toxin through the orbital septum and is one of the most recognized complications of treatment following glabella corrugator injections. The incidence is estimated to be 0.71%.^40^

#### Possible Solutions

The dose, placement, and depth of BoNTA need to be considered in order to solve potential frown treatment issues. BoNTA dose needs to be decreased for less bulky glabellar muscles, for example, female or Asian patients.^10-12^ A more conservative dose at the mid-pupillary point will be less likely to weaken the levator palpebrae superioris muscle and cause eyelid ptosis. The recommended placement, keeping a safe distance from the supraorbital margin to prevent eyelid and brow ptosis, as depicted in older publications, is problematic as it does not take into consideration the intersection points of the corrugator tail, which is usually lower (Figures 7, 8).^13,14^

In the glabella, the injection depth for the medial corrugator is typically deeper than the more superficial lateral insertion.^41^ The estimated depth is ∼4 mm according to the literature.^42^ In a recent study on cadaver and live ultrasound measurements, the authors estimated the depth of medial corrugator injection to be ∼4 mm.^42^ The lateral insertion can usually be observed during scowling as an area of skin puckering or “arrowing” when the eyebrow is retracted medially, which permits individualized treatment in terms of injection dose and placement.

**Figure 9.**
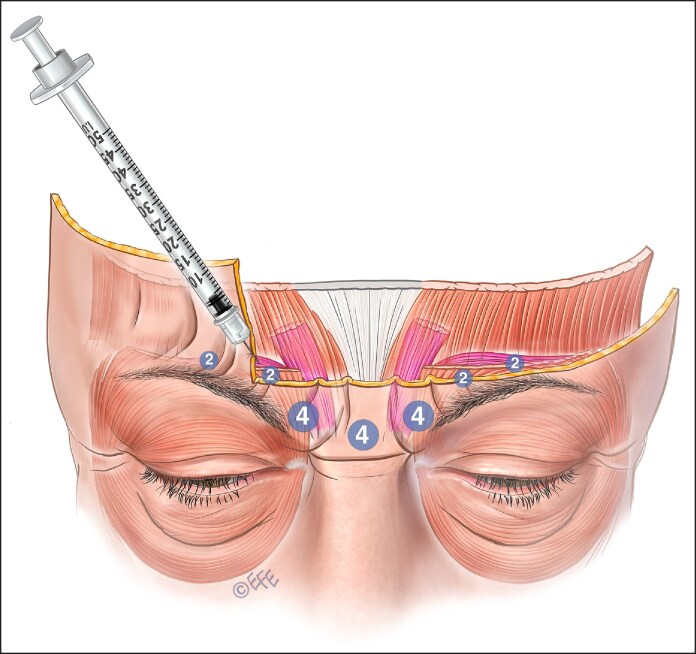
Lateral sites should be injected superficially, tangentially, with a smaller dose to avoid ptosis from unwanted diffusion to adjacent muscles such as the frontalis or levator palpebrae superioris.

A 5- to 7-point glabella injection pattern in a Caucasian is generally appropriate with the lateral, superficial insertion of the corrugator supercilii into the dermis of the forehead mirrored by the injections' depth. Not injecting laterally enough may allow function or even recruitment of lateral insertions of corrugator supercilii or medial OOc (Figure 8). Lateral injections should be superficial, tangential (not toward the eye), and with a smaller dose with gentle extrusion pressure (Figure 9).

#### Specific Remedies for Eyebrow Oddities: Mephisto/Spock

To correct a Spock eyebrow, treat the apex of muscle elevation on the frontalis with low-dose injections. Consideration can be given to injecting the relevant brow depressors (such as procerus, depressor supercilii, OOc, and orbicularis pars palpebralis).

#### Splaying of Eyebrows or Glabella Widening

This requires adequate patient counseling, explaining the mechanism, and gaining their acceptance. Consider dose reduction to the medial corrugator and suggest the patient does not pluck the medial eyebrow. Initiate topical bimatoprost if eyebrow hairs are sparse or use cosmetic brow enhancement techniques medially.

#### Eyelid Ptosis

Eyelid ptosis can be treated with α-adrenergic eye drops that induce contraction of the upper portion of the tarsal muscle, known as Müller's muscle. This can create a 1 to 2 mm elevation of the upper portion of the eyelid, which is usually sufficient to make the eyelids more symmetric. Injection of 1 unit (onabotulinum or equivalent) at the medial and lateral ends of the pretarsal orbicularis may also be considered.

#### Alternative Treatment Options

Surface-active procedures and devices can improve skin texture, as can superficially placed fillers; however, the latter carries a risk of intravascular occlusion. BoNTA remains the most effective treatment for glabellar scowl lines. Selected patients may benefit from surgical disruption of the corrugator muscles, although the procedure is invasive—either open or endoscopic—and may result in scarring, residual movement and asymmetry, and sensory nerve damage.

### Bunny Lines—The Role of the Levator Labii Superioris Alaeque Nasi

#### The Targeted Expression and Assessment

**Figure 10.**
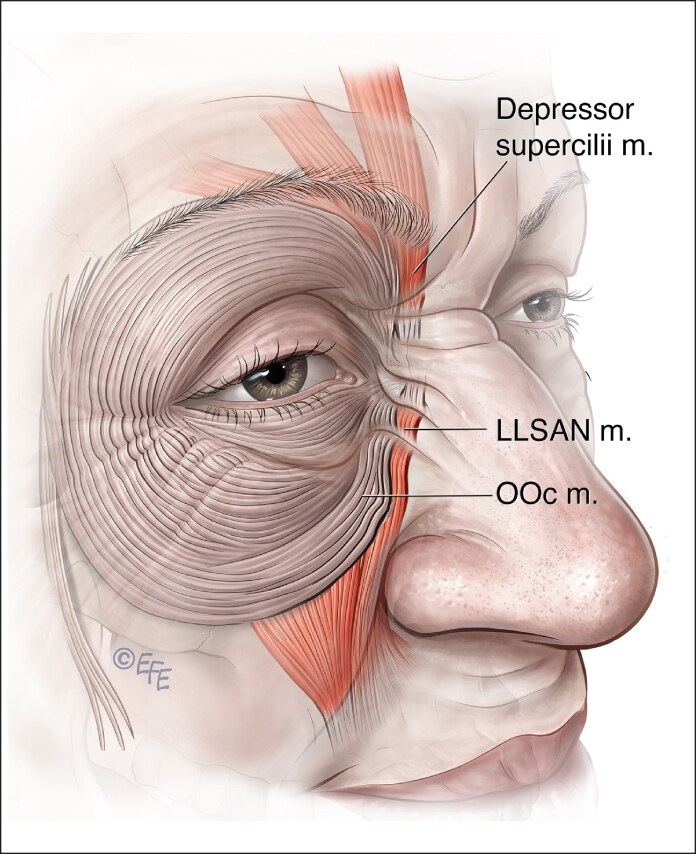
As shown in this diagram, in many circumstances, the depressor supercilii or orbicularis oculi is connected to the levator labii superioris alaeque nasi.

“Bunny” or “wolf” lines are colloquial terms for the oblique lines on the nasal sidewall that appear upon nose scrunching. They may occur on their own or in combination with smiling or frowning (Figure 10).

#### The Relevant Muscular Anatomy of the Region

Although initially thought to be caused by the action of the nasalis muscle, oblique wrinkles over the nasal sidewall are attributed to the superior origin of the LLSAN and medial inferior OOc.^43-45^ The depressor supercilii has now been shown to be continuous in close to 25% of cadavers with the LLSAN, and in 75% the depressor supercilii connects to either medial fibers of OOc or LLSAN.^46^ Medial brow depression should be assessed when injecting bunny lines (Figure 10).

#### Variations in Movement Patterns

Importantly, significant variabilities in muscle position and interaction exist, both between individuals and across ethnicities, making this a difficult area to target precisely with neuromodulators.^47^

#### On-Label or Standard Techniques

There is no on-label injection pattern described for the treatment of bunny lines.

#### Problems That May Arise

Hyperactive bunny line activity after glabella treatments tends to look very unnatural.

An injector should therefore determine whether bunny lines are created when frowning or smiling and consider incorporating this area into the treatment plan if necessary. Because of the action of LLSAN on the upper lip, it is also important to consider upper lip position and dynamic symmetry to avoid lengthening the upper lip when treating bunny lines. Keep the injection on the nose as medial as possible to limit this possibility.

#### The Possible Solutions

To ensure maximum effect, we recommend targeting the intersection between a transverse line at the level of the rhinion and a vertical line at the level of the medial canthus when treating bunny lines with 2 units of onabotulinum toxin or the equivalent to each side.^48^ The injection points should be kept toward the nose as much as possible to prevent overrelaxation of medial OOc, which may lead to worsening of medial eye bags (if present) or altering the natural elevation of the pretarsal orbicularis when smiling.

#### Alternative Therapies

There are options to treat static bunny lines, such as surface-active devices, but only botulinum toxin will affect movement in this region.

### The Crow's Feet (OOc): Not Just Lateral Canthal Lines

#### The Targeted Expression and Assessment

**Figure 11.**
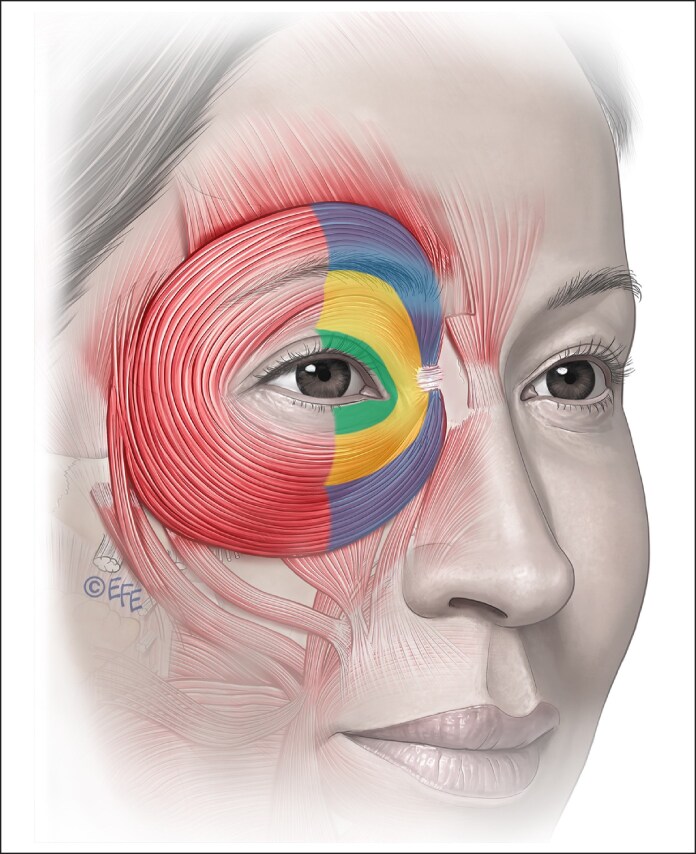
The orbicularis oculi is a concentric sphincter muscle arranged in concentric bands around the upper and lower eyelids. It is a large muscle and is present in the upper cheeks and lower forehead and brow. It is divided into 3 sections that are designated as pretarsal (green), preseptal (yellow), and orbital (blue) each with different functions.

There are multiple functions and potential targets in treating the OOc muscle (Figure 11, Table 4).^49^

**Table 4.** Targeted Expression Treating Orbicularis Oculi

Functions and potential targets	Intended result
Lateral canthal lines (crow's feet)	Targeting lateral orbicularis will reduce lateral canthal lines
Brow height	Targeting superolateral or depressor parts of orbicularis can elevate the brow
Ptosis	As the orbicularis oculi closes the eye, precise targeting of pretarsal orbicularis in the upper lid can alleviate ptosis^47^
Palpebral aperture	Targeting the upper and lower pretarsal orbicularis may widen the palpebral aperture
Bunny lines (medial crow's feet)	Targeting inferomedial orbicularis may reduce bunny lines

#### Relevant Muscular Anatomy of OOc

The OOc is a large, complex muscle and is divided into a number of parts with different functions.

The palpebral orbicularis is positioned between the lashes and the orbital margin and interacts with the orbital retaining ligament. It lacks subcutaneous tissue and separates from the dermis through direct contact with a thin fascial layer.

The pretarsal orbicularis is situated anterior and overlying the tarsal plate and provides lid support and functions in blinking and promoting tear flow.

The preseptal orbicularis is located between the pretarsal and orbital sections of OOc and aids in lid support, closure, and orbital fat retention.

The orbital orbicularis is located outside the orbital margin and is attached to the medial orbital margin, intersects with the orbital retaining ligament, and interdigitates with the frontalis and corrugator muscles. It is covered by increasing thickness of subcutaneous fat. The functions of the orbital section are very important in cosmesis as the responsible muscle for voluntary and forced eyelid closure, elevating the cheek, and supporting the overlying malar cheek fat and skin.

#### Variations in Movement Patterns

The orbicularis extends from above the brow to the base of the nose. Contraction creates wrinkles both perpendicular and parallel to muscle fibers. A thorough understanding of the muscles impacting brow and eyelid shape is essential for injection accuracy. They include the frontalis, OOc, corrugator supercilii, procerus, depressor supercilii, levator palpebrae superioris, and lower lid retractors, which can also influence wrinkle patterns.

Additionally, wrinkle appearance is influenced by factors such as skin thickness, elasticity, age, genetics, bone structure, and environmental factors. Considering these variables minimizes the risk of creating an imbalance.

#### On-Label Techniques

The only on-label BoNTA indication targeting the orbicularis is the treatment of the lateral canthal lines, or crow’s feet. The standard treatment protocol is 3 injection sites per side, in the lateral OOc muscle. The first point is often taught as 1.5 to 2 cm temporal to the lateral canthus, and the other 2 sites are 1 to 1.5 cm above and below this point. In general, 4 units of onabotulinum toxin A, or equivalent, are recommended at each site for a total of 12 units per side.^50^ In an early dose-ranging study, Lowe et al showed the preferred dose for 4-month longevity with onabotulinum toxin to be 12 units per side.^51^

#### The Problems That May Arise

Insufficient knowledge of the possible adverse effects when injecting the periorbital area can induce an array of unwanted outcomes.^52,53^

Gradual thinning of the dermis allows skin to wrinkle on animation because of a combination of age-related skin changes, photodamage, gravitational cheek descent, orbicularis laxity over the septum, globe descent caused by Whitnall's ligament, and infraorbital fat prolapse.

Unintended aging effects may occur when the support of the eyelid and malar fat pad is diminished by weakening the orbital OOc with BoNTA. This can also lead to a decrease in cheek fullness, as the volume of the orbicularis is reduced following BoNTA. In some cases, a flattened upper cheek, referred to as “shelfing,” may develop as a result of BoNTA injections in the lateral orbicularis, creating a disingenuous smile. Overinjection of the LLSAN or paralyzing the pretarsal orbicularis can result in paralytic ectropion or scleral show. Additionally, dry eye can arise from a reduction in the blink mechanism, lagophthalmos, or ectropion.

Decreased tear outflow and altered tear film stability may be linked to hypotonicity of the medial pretarsal fibers.

Unwanted fat bag development may occur when medial arrowing in the lower preseptal orbicularis is eliminated through low-dose BoNTA placement.

Medial brow ptosis can result if the frontalis is affected during injections targeting the corrugator and orbicularis in the medial brow to reduce vertical lines.

Finally, an unsuccessful lateral brow lift involving attempts at relaxation of the superolateral OOc with BoNTA may be attributed to a preexisting ptotic lateral brow, a common feature of aging.

#### The Possible Solutions

Awareness of potential issues is paramount. Prioritizing correction of total facial balance is crucial, rather than fixating on specific details with small areas and overlooking the bigger picture.

**Figure 12.**
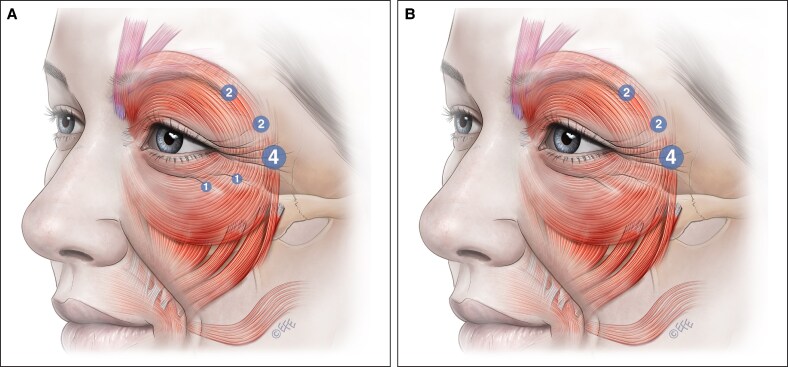
(A) Splitting the inferior third injection site into 2 sites with a reduced dose may also reduce adverse events. The lower sites should be injected superficially, tangentially and at the junction of preseptal and orbital orbicularis oculi to minimize weakening of the cheek elevating ability of orbital orbicularis avoiding shelfing and unnatural “fake” half smiles. (B) By removing the inferior third injection site, the risk of “shelfing” can be reduced. Impact to the Duchenne smile is reduced, and the patient can elevate the cheek and produce an honest, trustworthy appearance while reducing overall wrinkles. Alternative therapies can be employed for the lower rhytids.

Consider reducing the number of units or removing the third inferior injection site in the lower part of the OOc muscle inferior to the lateral canthus to minimize the impact on cheek elevation when smiling (Figure 12A, B). This part of the OOc is the primary cheek elevator in the face. The zygomaticus major and minor muscles predominantly elevate the corner of the lip. Minimizing treatment of this lower part of the OOc reduces the risk of producing a “half smile.”

Furthermore, it is recommended to avoid injections below the level of the superior zygomatic arch because this can impact the function of the zygomaticus major and/or minor muscles, resulting in ipsilateral lip ptosis.^54,55^

Focused communication and clear expectation setting are vital in the crow's feet BoNTA patient. A detailed explanation of expected outcomes and longevity of treatment should be discussed to ensure patient satisfaction. Patients who demand complete removal of the inferior rhytids may not understand the impact on their smile and their overall appearance.

#### Alternative Treatment Options

Alternative therapies include surgery, which is beyond the remit of this article. Judicious use of filler in the lateral OOc can support the area, add volume, and prevent skin folding, offering reasonable effectiveness against crow's feet.

Fractionated ablative and nonablative laser resurfacing, energy-based devices, mono-threads, and skin needling can stimulate collagen remodeling and reduce the appearance of static fine lines. Skin needling with insulin has been shown to improve acne scars and stria alba (stretchmarks), and some of the authors have replicated these results by reducing facial rhytids.^56,57^

Novel injectables, such as those incorporating stem-cell-derived exosomes and DNA-polynucleotide, are being utilized to improve skin quality and fine lines in this region but do not directly affect dynamic wrinkles that are because of muscle movement.^58-60^

## DISCUSSION

Injections of botulinum toxin may have beneficial effects on facial wrinkles and negative emotional expression. However, it may have a less-than-optimal effect on patients. This may occur because of incorrect placement, but this may not be the fault of the practitioner. On-label treatment may cause issues in some patients.

Assessment of the glabella, brow, crow's feet, and surrounding areas begins with keen observation at rest and maximum muscular contraction. A recommended protocol includes baseline photography or video at rest and maximum frown, documentation of injection points, and an optional 2- to 3-week postinjection review that serves as a record and a template for subsequent refinement of injection sites.

Treatments in the forehead may induce brow ptosis if on-label treatment across the forehead especially in the aged patient or those who are using the frontalis to chronically lift their brows and eyelids into their correct position. In the aged patient, the deterioration in skin quality because of photodamage, aging changes, loss of elasticity, and muscle tone are also likely to be factors in the decision making about the wisdom of forehead injection with neuromodulators. The etching of static wrinkles may mitigate against a meaningful result with injection of BoNTA alone.

The pattern of injection suggested by on-label techniques leads to injecting in areas where, in many patients, frontalis muscle does not exist, especially in high medial injection points (Figure 1).

Added to the effects of aging, the variation in anatomy that has been shown to exist should direct the optimal injection pattern. In 1 study, 3 main variations of the frontalis muscle were seen on cadaver dissection based on the extent of interdigitation between the 2 bellies in the midline. This may contribute to the downward slant in the eyebrows in some individuals.^61^ Three patterns were described in this article related to whether the frontalis muscle interdigitates or not. Pattern 1 was designated to those who interdigitate or decussate at the eyebrow attachment low down on the brow, with the 2 bellies of the frontalis diverging thereafter. Pattern 2 is quite an extensive decussation up until the mid-length of the frontalis bellies and then divergence. Pattern 3 is complete independence of the frontalis muscle bellies with no decussation at all. Because the aim of botulinum injection is to inject only where muscle is present, understanding the patient’s anatomical pattern is important for optimal results. In Patterns 1 and 3, it would be wasteful to inject medially where no muscle is present. Males and females appear to have similar points of dehiscence above the superior orbital rim in Pattern 2 but males have a bigger forehead and more receding hairline. Thus, females may benefit from a higher injection pattern of neuromodulators than males.^62^ In the days of imaging becoming more readily available, baseline scanning may be useful in defining a patient's variation to guide the best injection.

In the glabella region, on-label treatment for the frown complex requires a fixed number of injections positioned higher than optimal for effectively targeting the corrugator supercilii. A number of clinical patterns have been noted, termed V shaped, U shaped, converging arrows, inverted omega, and omega.^32^ However, it would appear that these do not correlate with underlying anatomical variations.^63^ The pattern seen clinically may instead be a mixture of skin quality and resistance effects that may be very individual. Even in these patterns, it may be best to inject in a standard way and assess the results after treatment, as often these patterns will still respond once the corrugator has been relaxed. In the glabella, it may be more pertinent to assess for the contribution of accessory muscles, such as LLSAN, OOc, and depressor supercilii rather than chasing patterns of the frown. The corrugator supercilii would appear constant in origin and insertion, and following this known anatomy and looking for the leaflets inserting into the skin just above the brow laterally might optimize the injection pattern.

Neglecting accessory muscles may result in incomplete treatment and recruitment of residual muscle activity, especially laterally, or recruitment of “bunny lines.” Treatment may also induce medial brow depression and lateral brow elevation producing the Mephistopheles appearance.

Anatomical variation is seen with OOc in its attachments and collaboration with other muscles in the production of certain movements like squinting or grimacing.^46^ It may connect with fibers of the depressor supercilii or LLSAN, inducing changes in the balance between facial elevators and depressors (Figure 10). In the OOc on-label therapy of the inferior orbital OOc, below the lateral canthus, suboptimal results may induce an inability to lift the cheek when smiling, rendering a fake or insincere smile and a physical appearance of shelfing.

The consensus of our group was that knowledge and utilization of anatomy should be paramount; however, anatomical variability and the individual ways that different patients use the same muscles need to be considered. Neither of these is optimally associated with standardized on-label injection methods.

For the forehead, assessment of why the lines are occurring is important. Botulinum toxin may not be the correct treatment or may need injecting in a different pattern to retain some muscle function. For the frown area, looking for small indentations from the pull of the muscle on the skin is the best way to work out where to inject. To keep the lateral injection points “safe,” a lower dose, superficial injection, and pointing the injection away from the eye are useful safety options. Assessing the patient's frown will determine which additional muscles require injection, such as the depressor supercilii, the LLSAN or elements of the OOc. For the OOc, avoid injecting higher doses in the inferior crow's feet, or even avoid this area entirely and inject closer to the septal area of the OOc.

Limitations to this include a paucity of relevant studies to support the opinions discussed here. We are suggesting shifting treatments away from on-label uses, with treatment placements discussed here reflecting the expert opinions of this panel.

## CONCLUSIONS

BoNTA injections can effectively reduce facial wrinkles and modulate negative facial expression. Achieving optimal outcomes requires a nuanced approach that accommodates individual anatomy and muscle dynamics. Although on-label protocols provide an approximate and generic guide, adapting injection points to address variability in anatomy and movement patterns is crucial to minimize complications, avoid odd-looking results, and enhance patient satisfaction. Ongoing education to reconcile muscular anatomy with facial expression, careful assessment, and a patient-centered approach will ensure the safe and effective use of BoNTA in aesthetic practice.

## Supplementary Material

ojaf032_Supplementary_Data
