# Supplementary material for: Botulinum Toxin: Surely, We Can Do Better? Optimizing Results Beyond On-Label Techniques and Teaching
Source: Aesthet Surg J Open Forum. 2025 Apr 30;7:ojaf032. doi: 10.1093/asjof/ojaf032 (PMC12205436; doi:10.1093/asjof/ojaf032)
Supplement: ojaf032_Supplementary_Data [file ojaf032_supplementary_data.zip › Supplementary Table_1.docx]

Supplementary Table 1: Expression and muscle involvement effect (forehead)

| Targeted expression | Effect | Targeted Muscle |
| --- | --- | --- |
| Horizontal forehead lines | Raising Eyebrows  Lowering hairline | Frontalis |
