# Supplementary material for: Botulinum Toxin: Surely, We Can Do Better? Optimizing Results Beyond On-Label Techniques and Teaching
Source: Aesthet Surg J Open Forum. 2025 Apr 30;7:ojaf032. doi: 10.1093/asjof/ojaf032 (PMC12205436; doi:10.1093/asjof/ojaf032)
Supplement: ojaf032_Supplementary_Data [file ojaf032_supplementary_data.zip › Supplementary Table_2.docx]

Supplementary Table 2: Issues with on-label treatment of frontalis muscle

| Problem | Avoidance |
| --- | --- |
| Brow ptosis | Avoid injecting frontalis in patients who have aged, low or unsupported brows |
| Frozen non-responsive forehead | Lessen botulinum dose or perhaps more superficial injection |
| Alteration in brow shape | After the injection method according to the patient's individual patterning. |
| Recruited or aberrant forehead lines superiorly or inferiorly | Alter injection pattern accordingly |
| Brow asymmetry | Assess pre-existing asymmetry and adjust for this. Consider selectively treating antagonistic depressors. |
