# Supplementary material for: Botulinum Toxin: Surely, We Can Do Better? Optimizing Results Beyond On-Label Techniques and Teaching
Source: Aesthet Surg J Open Forum. 2025 Apr 30;7:ojaf032. doi: 10.1093/asjof/ojaf032 (PMC12205436; doi:10.1093/asjof/ojaf032)
Supplement: ojaf032_Supplementary_Data [file ojaf032_supplementary_data.zip › Supplementary Table_3.docx]

Supplementary Table 3: Origin and insertion of Frown complex muscles

| Muscle | Shape / characteristics | Nuances of Origin | Nuances of insertion |
| --- | --- | --- | --- |
| Corrugator supercilii^28,29,30^ | Thin, small, rectangular muscle | On Bone  The medial end of the superciliary arch extending to the supraorbital notch | Inserts in the skin in multiple “leaflets” producing dimples just above the brow.  Interdigitates with frontalis and orbicularis oculi |
| Procerus^30^ | Pyramidal shaped muscle | Fascia and cartilage  Fascia of the superior nasal region and the superolateral nasal cartilage | The procerus muscle fibers run superiorly and merge with the frontalis muscle. Muscle fibers insert into the skin between the eyebrows. |
| Orbicularis oculi^31^ | Large sphincteric muscle divided into orbital and palpebral sections.  The palpebral divided into pre-septal and pre-tarsal components | Attaches to the medial canthal region medially and the lateral canthal region laterally.  With sphincteric muscles, origins and insertions are not individual concepts. | The orbicularis oculi subdivisions have a complex arrangement of attachments to the surrounding structures.  It is attached to bone superiorly and inferiorly via the orbicularis retaining ligament. The lower eyelid medial fibers may produce superior “bunny” lines on the nose whereas the fibers arching above the brows contribute to the lateral skin insertion points when frowning |
